# Plasmid-Chromosome Crosstalk in *Staphylococcus aureus*: A Horizontally Acquired Transcription Regulator Controls Polysaccharide Intercellular Adhesin-Mediated Biofilm Formation

**DOI:** 10.3389/fcimb.2021.660702

**Published:** 2021-03-22

**Authors:** Gabriella Marincola, Greta Jaschkowitz, Ann-Katrin Kieninger, Freya D.R. Wencker, Andrea T. Feßler, Stefan Schwarz, Wilma Ziebuhr

**Affiliations:** ^1^ Institute of Molecular Infection Biology, University of Würzburg, Würzburg, Germany; ^2^ Centre for Infection Medicine, Institute of Microbiology and Epizootics, Free University of Berlin, Berlin, Germany

**Keywords:** *Staphylococcus aureus*, biofilm regulation, PIA/*ica*, IcaR, horizontal gene transfer, plasmid-chromosome crosstalk

## Abstract

Livestock-associated methicillin-resistant *Staphylococcus aureus* (LA-MRSA) of clonal complex CC398 typically carry various antimicrobial resistance genes, many of them located on plasmids. In the bovine LA-MRSA isolate Rd11, we previously identified plasmid pAFS11 in which resistance genes are co-localized with a novel *ica*-like gene cluster, harboring genes required for polysaccharide intercellular adhesin (PIA)-mediated biofilm formation. The *ica* genes on pAFS11 were acquired in addition to a pre-existing *ica* locus on the *S. aureus* Rd11 chromosomal DNA. Both loci consist of an *icaADBC* operon and *icaR*, encoding a corresponding *icaADBC* repressor. Despite carrying two biofilm gene copies, strain Rd11 did not produce PIA and transformation of pAFS11 into another *S. aureus* strain even slightly diminished PIA-mediated biofilm formation. By focusing on the molecular background of the biofilm-negative phenotype of pAFS11-carrying *S. aureus*, we identified the pAFS11-borne *ica* locus copy as functionally fully active. However, transcription of both plasmid- and core genome-derived *icaADBC* operons were efficiently suppressed involving IcaR. Surprisingly, although being different on the amino acid sequence level, the two IcaR repressor proteins are mutually replaceable and are able to interact with the *icaA* promoter region of the other copy. We speculate that this regulatory crosstalk causes the biofilm-negative phenotype in *S. aureus* Rd11. The data shed light on an unexpected regulatory interplay between pre-existing and newly acquired DNA traits in *S. aureus.* This also raises interesting general questions regarding functional consequences of gene transfer events and their putative implications for the adaptation and evolution of bacterial pathogens.

## Introduction

**Figure 1:**
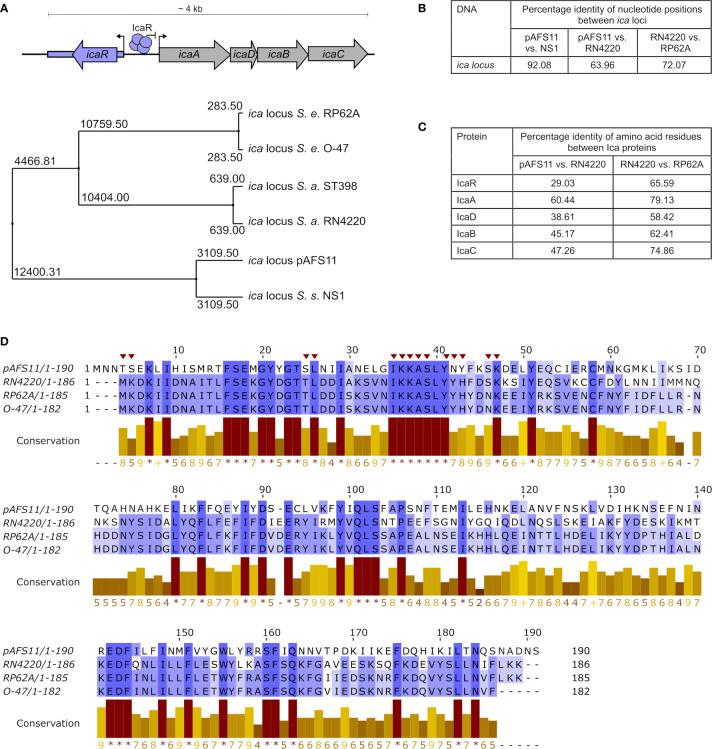
Conservation of pAFS11 *ica* locus. **(A)** TOP: schematic view of the organization of the *ica* locus including the IcaR tetramer and its function as repressor of transcription. BOTTOM: average distances between the *ica* loci of *S. epidermidis* O-47, *S. epidermidis* RP62A, *S. aureus* RN4220, *S. aureus* lineage ST398, *S. sciuri* NS1 and plasmid pAFS11. **(B)** Pairwise Alignment shown as percentage identity of nucleotide positions between the *ica* loci of pAFS11 vs. NS1, pAFS11 vs. RN4220 and RN4220 vs. RP62A. **(C)** Pairwise Alignment shown as percentage identity of amino acid residues between the Ica proteins of RN4220 vs. pAFS11 and RN4220 vs. RP62A. **(D)** Multiple sequence alignment of IcaR protein from pAFS11, RN4220, RP62A and O-47. Conservation is visualized as a histogram and a score is given for each column: conserved residues are indicated by ‘*’, and columns with residues, where all properties are conserved are marked with ‘+’. Putative *icaA* operon- interacting residues on IcaR in *S. epidermidis* are marked with a red triangle on top of the sequence (Jeng et al., 2008). All comparisons shown in **(A–D)** were calculated with the aid of Jalview (Waterhouse et al., 2009) from CLUSTAL Omega multiple sequence alignments (Madeira et al., 2019).

*Staphylococcus aureus* is a common human and animal pathogen, causing a wide range of clinical manifestations (Tong et al., 2015; Ballhausen et al., 2017). Due to the capability to readily acquire many different resistance genes, *S. aureus* and other staphylococcal species are regarded as pathogens of concern for public health (Foster, 2017; Lakhundi and Zhang, 2018). Thus, methicillin-resistant *S. aureus* (MRSA) and coagulase-negative staphylococci (MR-CoNS) are among the most common causes of healthcare-associated infections (Lee et al., 2018a; Becker et al., 2020). In this respect, the ability to form biofilms on the inert surfaces of medical devices is considered as important pathomechanism that contributed to the establishment of staphylococci as notorious nosocomial pathogens (Heilmann et al., 2019; Becker et al., 2020; Schilcher and Horswill, 2020). Biofilms are understood as bacterial communities that adhere to surfaces by encasing into a self-produced extracellular polymeric matrix (Costerton et al., 1999). The staphylococcal biofilm matrix may contain exopolysaccharides (Heilmann et al., 1996) and proteins (Rohde et al., 2005) as well as extracellular (e)DNA (Qin et al., 2007) [for a recent review see reference (Schilcher and Horswill, 2020)]. The key exopolysaccharide component of staphylococcal biofilms is PIA (polysaccharide intercellular adhesin), a beta-1,6 linked N-acetyl glucosaminoglycan, whose synthesis enzymes are encoded by the *ica* (intercellular adhesin) locus [recently reviewed in (Nguyen et al., 2020)]. PIA/*ica* was originally discovered in *Staphylococcus epidermidis* and was later also detected in *S. aureus* and other staphylococcal species (Heilmann et al., 1996; Mack et al., 1996; Cramton et al., 1999). Interestingly, *ica* locus homologs also exist in phylogenetically unrelated bacteria such as *Escherichia coli* (Wang et al., 2004), suggesting an eminent role of the factor in the evolution of bacterial biofilm functions. In these organisms, PIA is also often referred to as PNAG (poly-1,6-N-acetylglucosamine). The staphylococcal *ica* locus consists of two divergently oriented transcription units, one comprising the *icaADBC* operon (encoding the enzymes required for PIA synthesis) and the other harboring *icaR* which codes for a transcription factor of the TetR family (**Figure 1A**). IcaR, for which the crystal structure was solved, binds to a region upstream of *icaA* and represents a potent repressor of *icaADBC* operon transcription (Conlon et al., 2002a; Jefferson et al., 2004; Jeng et al., 2008). Regulation of the *ica* locus is highly complex and a plethora of environmental cues are known to influence PIA production many of which either directly or indirectly influencing *icaR* transcription (Conlon et al., 2002b; Cerca et al., 2008; Fey and Olson, 2010; Cue et al., 2012; Hoang et al., 2019; Nguyen et al., 2020). Expression of *icaR* is further controlled post-transcriptionally through RNA-mediated mechanisms that influence stability and translation of the *icaR* mRNA, with direct consequences for PIA production and biofilm formation (Ruiz de los Mozos et al., 2013; Rochat et al., 2018; Bronesky et al., 2019; Lerch et al., 2019; Schoenfelder et al., 2019). While nearly all *S. aureus* genomes carry the *ica* locus, distribution of the gene cluster among *S. epidermidis* and other CoNS species is more diverse and often associated with distinct clonal lineages (Kozitskaya et al., 2005; Conlan et al., 2012; Thomas et al., 2014; Méric et al., 2015; Méric et al., 2018; Lee et al., 2018b; Espadinha et al., 2019). The *ica* locus is usually located in the bacterial chromosomal DNA in all staphylococcal species. Previously, however, we detected an *ica* gene cluster of unknown genetic origin on plasmid pAFS11 in the bovine MRSA isolate *S. aureus* Rd11 (Feßler et al., 2017). *S. aureus* Rd11 is a livestock-associated (LA)-MRSA strain of sequence type ST398, a clonal lineage known for its potential to carry a broad range of both common and novel antibiotic resistance genes, many of which located on plasmids (Kadlec et al., 2012; Feßler et al., 2018). On pAFS11, antimicrobial and heavy metal resistance genes were found to be co-localized with a novel *ica* gene cluster. The *ica* locus on pAFS11 differed both on nucleotide and protein levels from the copy in the *S. aureus* Rd11 chromosome, and initial analyses (*i.e.* BLAST searches against the entire non-redundant sequence collections at NCBI) suggested that the plasmid-borne *ica* locus might have its origin in the CoNS species *Staphylococcus sciuri* [recently re-classified as *Mammaliicoccus sciuri* (Madhaiyan et al., 2020)] (Feßler et al., 2017). The mosaic structure of pAFS11 further suggests that the plasmid arose by a series of recombination events and was acquired by *S. aureus* Rd11 through horizontal gene transfer (HGT). As a result, *S.* *aureus* Rd11 carries two *ica* loci. Surprisingly, however, the strain did not produce biofilm when tested in standard tissue culture plate assays. Also, transformation of the pAFS11 plasmid into another *S. aureus* strain did not prompt biofilm formation, but even slightly reduced it (Feßler et al., 2017). In this study, we address the molecular mechanism underlying the biofilm-negative phenotype of pAFS11-bearing *S. aureus*. We identified an unexpected IcaR-mediated regulatory crosstalk between the plasmid-borne and chromosomally encoded *ica* loci, resulting in downregulation of biofilm formation. We discuss these findings in the context of co-evolution of virulence and resistance traits and raise the question of how genes newly acquired by HGT might become integrated into the regulatory network of host bacteria.

## Materials and Methods

### Sequence Alignments and Data Base Searches

Alignments of the nucleotide sequences of *ica* loci as well as that of amino acid sequences of Ica proteins from different species were performed with CLUSTAL Omega multiple sequence alignments (https://www.ebi.ac.uk/Tools/msa/clustalo) (Madeira et al., 2019). Average distance between the *ica* loci and pairwise alignments were calculated with the aid of Jalview (Waterhouse et al., 2009). Strains and sequences included into the analyses comprised *S. epidermidis* RP62A (accession no. NC_002976), *S. epidermidis* O-47 (accession no. CP040883), *S. aureus* ST398 (accession no. AM990992.1), *S. aureus* RN4220 (accession no. AFGU01000118.1), *S. sciuri* NS1 (accession no. LDTK01000031.1) and plasmid pAFS11 (accession no. FN806789.3). Data base queries with nucleotide and protein sequences were performed using the Basic Local Alignment Search Tool available at the National Center for Biotechnology Information (NCBI) (https://blast.ncbi.nlm.nih.gov/Blast.cgi).

### Plasmid and Strain Construction

Strains, plasmids and oligonucleotides used for this work are listed in **Tables 1** and **2**, respectively.

**Table 1:** Strains and plasmids.

	Description	Reference
**Strains**		
***E. coli***		
DC10B	*E. coli* for plasmid transformation into staphylococci	(Monk et al., 2012)
***S. aureus***		
RN4220	Restriction-deficient *S. aureus* strain	(Nair et al., 2011)
Rd11	LA-MRSA carrying pAFS11 plasmid	(Feßler et al., 2017)
N2	RN4220 transformed with pAFS11	(Feßler et al., 2017)
GAM20	RN4220 transformed with pGM10 (p*ica*_pAF_)	This work
GAM28	RN4220 transformed with pGM11 (p*ica*_pAF__Δ*icaR*_pAF_)	This work
AK18	RN4220 transformed with pAK17	This work
GAM30	RN4220 Δ*ica*	This work
GAM33	GAM30 (RN4220 Δ*ica*) transformed with pGM10 (p*ica*_pAF_)	This work
GAM35	GAM30 (RN4220 Δ*ica*) transformed with pGM11 (p*ica*_pAF__Δ*icaR*)	This work
GAM42	GAM30 (RN4220 Δ*ica*) transformed with pGM12 (p*ica*_RN_)	This work
GAM44	GAM30 (RN4220 Δ*ica*) transformed with pGM13 (p*ica*_RN__Δ*icaR*)	This work
GAM46	GAM30 (RN4220 Δ*ica*) transformed with pGM14 (p*ica*_RN__Δ*icaR*_*icaR*_pAF_)	This work
GAM49	GAM30 (RN4220 Δ*ica*) transformed with pGM15 (p*ica*_pAF__Δ*icaR*_*icaR*_RN_)	This work
GAM57	GAM30 (RN4220 Δ*ica*) transformed with pGM16 (p*ica*_pAF(A***)_)	This work
GAM59	GAM30 (RN4220 Δ*ica*) transformed with pGM17 (p*ica*_pAF(A***)__Δ*icaR*)	This work
GAM61	GAM30 (RN4220 Δ*ica*) transformed with pGM18 (p*ica*_pAF(A***)__Δ*icaR*_*icaR*_RN_)	This work
GAM63	GAM30 (RN4220 Δ*ica*) transformed with pGM19 (p*ica*_pAF(B***)_)	This work
GAM65	GAM30 (RN4220 Δ*ica*) transformed with pGM20 (p*ica*_pAF(B***)__Δ*icaR*)	This work
GAM67	GAM30 (RN4220 Δ*ica*) transformed with pGM21 (p*ica*_pAF(B***)__Δ*icaR*_*icaR*_RN_)	This work
**Others**		
RP62A	*S. epidermidis* biofilm positive reference strain	(Gill et al., 2005)
TM300	*S. carnosus* biofilm negative reference strain	(Que et al., 2005)
**Plasmids**		
pAFS11	Original plasmid isolated from Rd11	(Feßler et al., 2017)
***icaADBC* mutant construction**		
pBASE6	Suicide mutagenesis vector	(Geiger et al., 2012)
pAK17	pBASE carrying *ica*_RN_ flanking region for *ica* deletion	This work
***ica* complementation**		
pRB473	Staphylococcal shuttle vector	(Brückner et al., 1993)
pGM10	pRB473 with *ica* operon from pAFS11 (*ica*_pAF_)	This work
pGM11	pRB473 with *ica*_pAF__Δ*icaR*	This work
pGM12	pRB473 with *ica* operon from RN4220 (*ica*_RN_)	This work
pGM13	pRB473 with *ica*_RN__Δ*icaR*	This work
pGM14	pRB473 with *ica*_RN__Δ*icaR*_*icaR*_pAF_ (“crosstalk plasmid”)	This work
pGM15	pRB473 with *ica*_pAF__Δ*icaR*_*icaR*_RN_ (“crosstalk plasmid”)	This work
pGM16	pRB473 with *ica*_pAF_ with palindrome A mutated (*ica*_pAF(A***)_)	This work
pGM17	pRB473 with *ica*_pAF(A***)__Δ*icaR*	This work
pGM18	pRB473 with *ica*_pAF(A***)__Δ*icaR*_*icaR*_RN_	This work
pGM19	pRB473 with *ica*_pAF_ with palindrome B mutated (*ica*_pAF(B***)_)	This work
pGM20	pRB473 with *ica*_pAF(B***)__Δ*icaR*	This work
pGM21	pRB473 with *ica*_pAF(B***)__Δ*icaR*_*icaR*_RN_	This work

“***” symbolized mutated palindromes.

**Table 2:** Oligonucleotides.

Purpose	Template	Name	Sequence
**qRT-PCR**			
*gyr*	–	GM027	ACGGATAATTATGGTGCTGGGC
		GM028	TGCAAACCTCTCTCTGAAGTCG
*icaA_RN_*	–	GM020	AACAGAGGTAAAGCCAACGC
		GM021	ATGGTGCATCTTGATCAACG
*icaA_pAF_*	–	GM016	ATTTGATGTGTGTCGATGCAG
		GM017	TCCCTGTTACTGCTCCGATTG
*icaR_pAF_*	–	GM018	ATGTTTGTATACGGATGGCTTT
		GM019	ATCAGCGTTTGACTGATTCG
***ica* deletion mutant**			
pAK17	RN4220	Flank_A_SLIC	GATCTGTCGACGATAACAGATACTATTGGAGATACT
		Flank_A_rev	ATTGGCATTGGTAAATCATGACATAGGCGCTT
		Flank_B_rev	ATGATTTACCAATGCCAATGGGAGTGGGACA
		Flank_B_SLIC	GCATGCAAGCTTGATAGGAACACCACATAATGGTA
	pBASE6	SLIC_pBASE_R	TATCGTCGACAGATCTGCGCG
		SLIC_pBASE_F	TCAAGCTTGCATGCCTGCAGAA
Deletion Confirmation		GM176	TTGCTAAAACAATACCAACAATA
		GM177	AAGGTAATCATGACAATATGAT
**Complementation plasmids**			
pGM10	pRB473	SLIC_pRB473_R	GTCGACTCTAGAGGATCCCCGG
		SLIC_pRB473_F	CTGCAGGCATGCAAGCTTGGATTCT
	pAFS11	GM154	CTTGCATGCCTGCAGACAGAAGACTCCTTTTTGTT
		SLIC_icaCSc_R	TCCTCTAGAGTCGACGAAGATAAACATTACCTATA
pGM12	pRB473	SLIC_pRB473_R	GTCGACTCTAGAGGATCCCCGG
		SLIC_pRB473_F	CTGCAGGCATGCAAGCTTGGATTCT
	RN4220	GM178	CTTGCATGCCTGCAGATCACATAGGCGCTTATCAAT
		GM179	TCCTCTAGAGTCGACTACGAAGTTTAAATGTGCAAT
pGM11	pGM10	GM156	GAGGCAAATGAAGATAATTCATAAAAACCTATAATGA
		GM155	GGTTTTTATGAATTATCTTCATTTGCCTCCTTTACTA
		GM157	AGGCAGTTATTGGTGCCCTTAAACG
		pRB473_MCS_F	CGTTTAAGGGCACCAATAACTGCCT
pGM13	pGM12	GM182	GTAGGGGGTTATAAAAATTTTTGTTACTAGTTTGTAATA
		GM183	AAACTAGTAACAAAAATTTTTATAACCCCCTACTGAAAATTA
		GM157	AGGCAGTTATTGGTGCCCTTAAACG
		pRB473_MCS_F	CGTTTAAGGGCACCAATAACTGCCT
pGM14	pAFS11	GM186	GTAGGGGGTTATAAAAAGTGAATAATACATCTGAGAAACTC
		GM187	ACAAACTAGTAACAAAAATTATGAATTATCAGCGTTTGACT
	pGM212	GM184	TTTTTATAACCCCCTACTGAAAATTAA
		GM185	TTTTTGTTACTAGTTTGTAATAATTAA
pGM15	RN4220	GM196	AGTAAAGGAGGCAAATGAATTGAAGGATAAGATTATTGATA
		GM197	TGTCATTATAGGTTTTTATTTCTTCAAAAATATATTTAGT
	pGM10	GM198	AAACCTATAATGACACGCCATA
		GM199	TTCATTTGCCTCCTTTACTACCTATGAATA
pGM16/pGM17/pGM18	pGM10/pGM11/pGM15	GM202	ATAGTATATCtaaaagtAAGAAAAAGGCAATGCGTTA
		GM201	acttttaGATATACTATTTTTACAAACTACCG
		GM157	AGGCAGTTATTGGTGCCCTTAAACG
		pRB473_MCS_F	CGTTTAAGGGCACCAATAACTGCCT
pGM19/pGM20/pGM21	pGM10/pGM11/pGM15	GM203	aagcaatGGGAGAAAATTATGAAAATTTTATTA
		GM204	TTTTCTCCCattgcttCGGTAGTTTGTAAAAATAGTA
		GM157	AGGCAGTTATTGGTGCCCTTAAACG
		pRB473_MCS_F	CGTTTAAGGGCACCAATAACTGCCT

#### Construction of a Markerless *icaADBC* Mutant

The markerless *ica* mutant was obtained *via* allelic replacement with inducible counter-selection using the pBASE6 shuttle vector (Bae and Schneewind, 2006; Geiger et al., 2012). pBASE6 vector was linearized using primers SLIC_pBASE_R and SLIC_pBASE_F. Total deletion was achieved by overlapping PCR using as template gDNA from RN4220 with primers Flank_A_SLIC together with Flank_A_rev and Flank_B_rev together with Flank_B_SLIC. The amplicon was introduced into the linearized pBASE6 vector using the *in vivo E. coli* cloning (iVEC) method (Nozaki and Niki, 2019). The resulting plasmid (pAK17) was transformed into the restriction-deficient strain RN4220. Mutagenesis was performed as described elsewhere (Bae and Schneewind, 2006). The deletion was verified by PCR with oligonucleotides spanning the deletion region.

#### Construction of Complementation Plasmids

All plasmids were created following the iVEC method (Nozaki and Niki, 2019). As iVEC turned out to be more efficient in the presence of buffer, the ligation buffer from the QuickLigation™ Kit (NEB, #M2200S) was added to the reactions. Sanger sequencing was used to verify accuracy of all plasmids. To create plasmids pGM10 and pGM12, the *ica*_pAF_ and *ica*_RN_ operons were amplified from pAFS11 and RN4220, respectively, and introduced in the linearized pRB473. Deletion of *icaR* coding regions from plasmids pGM10 and pGM12 (resulting in plasmids pGM11 and pGM13, respectively) was achieved by overlapping PCRs which amplified the respective vectors in two fragments that overlapped in the *icaR* deletion and multiple cloning site regions. Of note, this approach left putative *icaR* 5’ and 3’ untranslated regions (UTRs) intact which might be involved in post-transcriptional regulation of *icaR*. *icaR*_pAF_ was amplified from pAFS11 and introduced in the linearized pGM12, resulting in plasmid pGM14. *icaR*_RN_ was amplified from RN4220 and introduced in the linearized pGM10, resulting in plasmid pGM15. The vectors containing mutated sequences which alter the palindromes (pGM16 to pGM21) were generated by PCR site-directed mutagenesis amplifying the original vectors (see **Table 2** for details) in two fragments that overlapped in the region containing the mutated palindrome and in the multiple cloning site region. In **Table 2**, the mutated palindrome sequences are shown as lower-case characters in the primer sequences.

### Preparation of Total RNA and qRT-PCR

Total RNA of bacteria was isolated as described previously (Lerch et al., 2019). Briefly, RNA was precipitated with 1x volume isopropanol (Sigma-Aldrich, #I9516) for 10 minutes at room temperature. Pelleted RNA was washed with 70 % ethanol and solved in RNase‐free double-distilled water (ddH_2_O). The transcript abundance of *icaA*_pAF_, *icaR*_pAF_ and *icaA*_RN_ from three independent experiments was determined by real-time qRT-PCR. Thus, 5 µg of each RNA sample was treated with DNaseI (Thermofisher, #AM2235) for 45 min at 37°C and the reaction was stopped by phenol/chloroform/isoamylalcohol extraction (25:24:1, Carl Roth GmbH, #X985.2) with the aid of PLG heavy tubes (5 Prime, #2302830). RNA was precipitated overnight at -20°C with 4.67x volume ethanol/3M sodium acetate pH 6.5 (Thermofisher, #AM9740) (30:1 mix). Pelleted RNA was washed with 70% ethanol, dissolved in 30 µl RNase‐free ddH_2_O and diluted 1:10. To check for efficiency of DNA digestion, a PCR was set up with the same amount of RNA (1 µl of 1:10) and same primers used for qRT-PCR. One-step qRT–PCR was performed using an amplification kit with SYBR Green (Power SYBR™, Green RNA-to-CT™ 1-Step Kit; Thermofisher, # 4389986) with the primers listed in **Table 2** and run on Biorad CFX according to the manufacturer’s instructions. Transcript abundance was calculated using a logarithmic dilution series of one sample to generate a standard curve for each gene. Relative quantification of the genes of interest was expressed in relation to the expression of the constitutive reference gene gyrase B (*gyrB*). The means were calculated from three biological replicates run in technical duplicates. Statistical analysis was performed using one-way ANOVA by employing the GraphPad Prism software package.

### Biofilm Assay

Biofilm formation was tested on 96-well, polystyrene tissue culture plates (Greiner Bio-One, # 655180) as described previously (Christensen et al., 1985), using Trypticase™ Soy Broth (BD BBL™, #211768) supplemented with 4% NaCl as growth medium. *S. epidermidis* RP62A and *S. carnosus* TM300 were used as positive and negative controls, respectively. For strains carrying resistance genes, antimicrobial agents were used at the following concentrations: 25 µg ml^−1^ erythromycin (for both overnight and day culture) and 30 µg ml^-1^ (for overnight culture) or 10 µg ml^-1^ (for day culture) chloramphenicol. Bacterial overnight cultures were freshly diluted to OD_600_ of 0.05 and 200 µl filled in each well (two technical replicates per strain). To distinguish between total, protein and PIA matrix-mediated biofilm production, three tissue culture plates were set up in parallel and incubated at 30°C for 18 h. Cultures were then discarded and adherent cells washed twice with 1x PBS buffer. The control plate for measuring the total biofilm was dried and heat-fixed at 65°C for 1 h. To discern between PIA- and protein- mediated biofilm, biofilms were either treated with 1 mg ml^−1^ proteinase K (Merck, #1245680500) for 4 h at 37°C or 40 mM NaIO_4_ (Carl Roth GmbH, #2603.1) for 24 h at 4°C. Afterwards the plates were washed with 1x PBS, dried and heat-fixed. All three plates were stained with 10 mg ml^−1^ crystal violet (Merck, #115940) for 2 min, washed twice with double-distilled water before measuring the absorbance at 492 nm (ELISA plate reader, Multiskan Ascent). The means were calculated from three biological replicates. Statistical analysis was performed using one-way ANOVA by employing the GraphPad Prism software package.

## Results

### The Two *ica* Locus Copies in *S. aureus* Rd11 Are of Different Genetic Origin

We previously reported that database searches against the entire non-redundant nucleotide collection at NCBI (including whole-genome shotgun contigs) returned similarities of pAFS11 to an *ica*-like gene cluster present in some *S. sciuri* isolates [now *M. sciuri* (Madhaiyan et al., 2020)] (Feßler et al., 2017). Of note, the putative *ica* locus on pAFS11 was found to differ on nucleotide level from *ica* sequences present in *S. aureus* and *S. epidermidis* (Feßler et al., 2017). For further phylogenetic analysis, we therefore performed multiple sequence alignments of *icaR/icaADBC* nucleotide sequences from two *S. aureus* (*i.e.* RN4220 and ST398, to which Rd11 belongs) and two *S. epidermidis* (*i.e.* RP62A and O-47) strains as well as from the pAFS11 *ica* locus (referred to as *ica*_pAF_ hereafter). Finally, based on the nucleotide BLAST query results, an *ica*-like locus from the CoNS species *S. sciuri* was included into the analysis as well. Average distances were calculated from the alignment data and the tree displayed in **Figure 1A** illustrates that *ica*_pAF_ is most distantly related to the two *S. aureus*-derived *ica* loci. In addition, the two *ica* loci from *S. epidermidis* are highly divergent from *ica*_pAF_, while they are closer related to *ica* from *S. aureus*. Interestingly, however, the *ica*_pAF_ nucleotide sequence is closely related to the *ica*-like locus from *S.* *sciuri* strain NS1 (**Figures 1A, B**). The data suggest that the two *ica* loci present in Rd11 are of different genetic origin, with *ica*_pAF_ most likely being derived from another species for which *S.* *sciuri* (*M. sciuri*) is a putative candidate. Although, as expected, interspecies conservation on the nucleotide level was found to be low (**Figure 1B**), the *ica*_pAF_ genes translate into amino acid sequences that are identified by the BLASTP algorithm as Ica-associated proteins with (again) some sequence differences between species. Thus, **Figure 1C** shows the percentage of identical amino acid positions upon pairwise alignments of Ica proteins from *ica*_pAF_, *S. aureus* RN4220 and *S. epidermidis* RP62A. While comparisons between RN4220 and RP62A revealed high conservation of *S. aureus* and *S. epidermidis* Ica proteins, identical amino acid positions were much lower between *ica*_pAF_ and *S. aureus*-derived Ica proteins. In this respect, IcaR was the protein with the lowest conservation (29%), indicating that the two IcaR repressor proteins harbored by *S. aureus* Rd11 differ significantly on the protein sequence level (**Figure 1C**). Despite this apparent divergence, IcaR_pAF_ exhibits a number of amino acid residues (marked by asterisks in the conservation histogram in **Figure 1D**) that are highly conserved in IcaR proteins of *S. aureus* and *S. epidermidis* as well (**Figure 1D**). These include the putative *icaA* operator-interacting residues shown for IcaR *S. epidermidis* (Jeng et al., 2008) (marked with red triangles on top of the sequence in **Figure 1D**).

### Plasmid pAFS11 Has a Negative Effect on *S. aureus* PIA-Mediated Biofilm Formation

**Figure 2:**
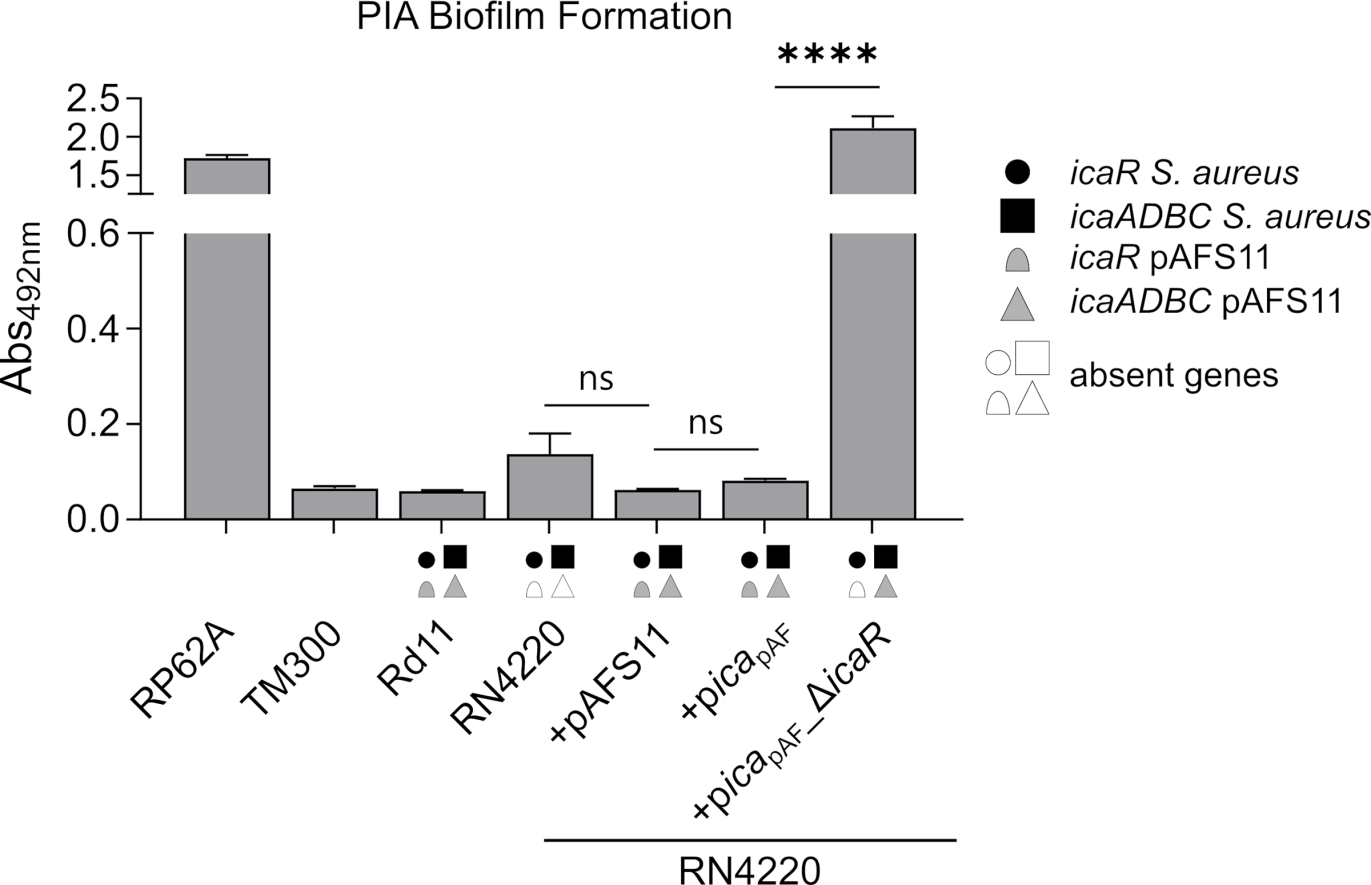
Effect of pAFS11 on PIA biofilm formation. Analysis of PIA biofilm production by static 96-well microtiter plate biofilm assays of Rd11, RN4220 and RN4220 transformed with plasmid pAFS11 or with a plasmid carrying the whole *ica* operon from pAFS11 (+p*ica*_pAF_) or with *icaR* deletion (+p*ica*_pAF__Δ*icaR*). RP62A served as positive control, TM300 as negative control. The means were calculated from three biological replicates run in duplicates. The *ica* genes distinctive for each strain are depicted as symbols, with filled symbols indicating presence and empty symbols indicating absence of a given gene (as indicated in the legend). The entire data sets on total, protein and PIA biofilm formation can be found in **Supplementary Figure S1**. Statistical analysis was performed using one-way ANOVA by employing the GraphPad Prism software package. ns: *P* = 0.1234; ****P < 0.0001.

**Figure 3:**
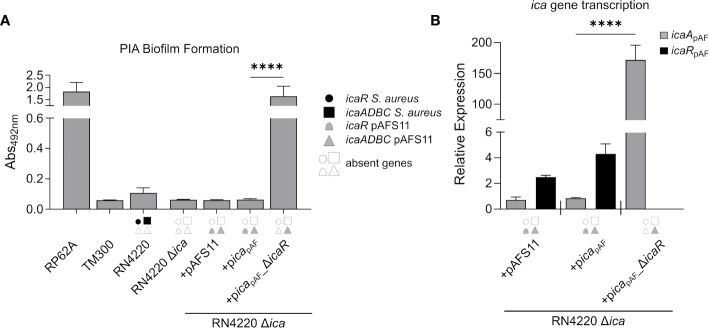
The *ica* genes of pAFS11 lead to biofilm formation. **(A)** Analysis of PIA biofilm production by static 96-well microtiter plate biofilm assays of strain RN4220 wild type and Δ*ica* alone or complemented with plasmid pAFS11 or with a plasmid carrying the whole *ica* operon from pAFS11 (+p*ica*_pAF_) or with *icaR* deletion (+p*ica*_pAF__Δ*icaR*). RP62A served as positive control, TM300 as negative control. The entire data sets on total, protein and PIA biofilm formation can be found in **Supplementary Figure S1**. **(B)** Quantification of *icaA*_pAF_ and *icaR*_pAF_ transcripts by qRT-PCR of strains from **(A)**. The graph displays relative mRNA amounts using *gyrB* expression as reference. **(A, B)** The *ica* genes distinctive for each strain are depicted as symbols, with filled symbols indicating presence and empty symbols indicating absence of a given gene (as indicated in the legend). The means were calculated from three biological replicates run in duplicates. Statistical analysis was performed using one-way ANOVA by employing the GraphPad Prism software package. ns: *P* = 0.1234; ****P < 0.0001.

**Figure 4:**
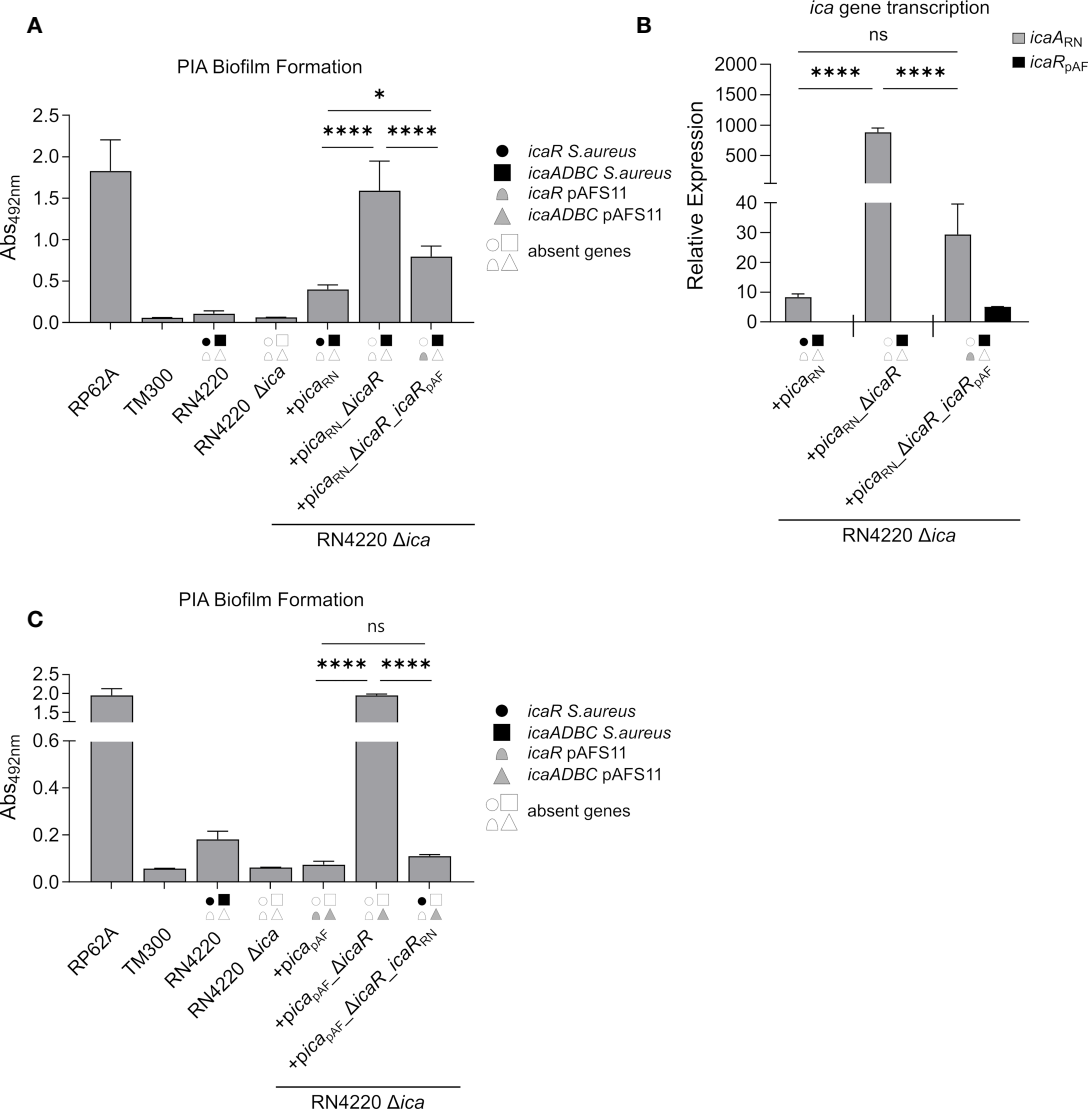
IcaR_pAF_ controls expression of *ica* genes from RN4220 and *vice versa*. **(A)** Analysis of PIA biofilm production by static 96-well microtiter plate biofilm assays of strain RN4220 wild type and Δ*ica* alone or complemented with a plasmid carrying the *ica* operon of RN4220 (+p*ica*_RN_) or with *icaR* deletion (+p*ica*_RN__Δ*icaR*) or with *icaR*_pAF_ instead of *icaR*_RN_ **(+p*ica*_RN__Δ*icaR_icaR*_pAF_). The entire data sets on total, protein and PIA biofilm formation can be found in **Supplementary Figure S1**. **(B)** Quantification of *icaA*_RN_ and *icaR*_pAF_ transcripts by qRT-PCR of complemented strains from **(A)**. The graph displays relative mRNA amounts using *gyrB* expression as reference. **(C)** Analysis of PIA biofilm production by static 96-well microtiter plate biofilm assays of RN4220 wild type and Δ*ica* alone or complemented with the *ica* operon of plasmid pAFS11 on a working plasmid (+p*ica*_pAF_) or the *ica* operon of plasmid pAFS11 with *icaR* deletion (+p*ica*_pAF__Δ*icaR*) or with *icaR*_RN_ instead of *icaR*_pAF_ (+p*ica*_pAF__Δ*icaR_icaR*_RN_). **(A, C)** RP62A served as positive control, TM300 as negative control. **(A–C)** The *ica* genes distinctive for each strain are depicted as symbols, with filled symbols indicating presence and empty symbols indicating absence of a given gene (as indicated in the legend). The means were calculated from three biological replicates run in duplicates. Statistical analysis was performed using one-way ANOVA by employing the GraphPad Prism software package. ns: *P* = 0.1234; *P = 0.0332; ****P < 0.0001.

**Figure 5:**
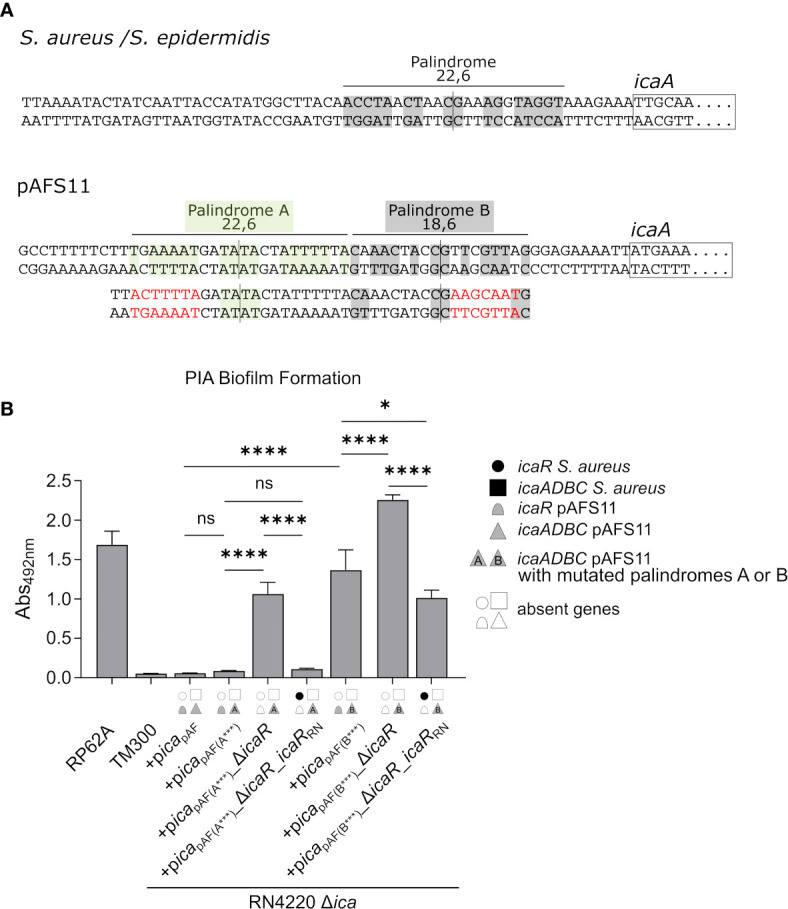
IcaR proteins from *S. aureus* and from pAFS11 require a palindrome on the *icaA*_pAF_ operator for action. **(A)** TOP: Known palindrome sequence on *S. aureus*/*S. epidermidis* is shown at the top of the panel (Jeng et al., 2008). 22,6 stands for a palindrome which is 22 nt long and carries six mismatches. BOTTOM: Putative palindromes A and B on *icaA*_pAF_ operator are shown and their characteristics are reported. The nucleotide mutations introduced are shown in red below the wild type sequence alongside with the resulting palindrome perturbations. **(B)** Analysis of PIA biofilm production by static 96-well microtiter plate biofilm assays of strain RN4220 Δ*ica* complemented with the *ica* operon from pAFS11 wild type (+p*ica*_pAF_) or with deletion of *icaR*_pAF_ (+p*ica*_pAF__Δ*icaR*) or with *icaR*_RN_ instead of *icaR*_pAF_ (+p*ica*_pAF__Δ*icaR_icaR*_RN_), carrying a mutated palindrome A (*ica*_pAF(A_***_)_) or B (*ica*_pAF(B_***_)_). The *ica* genes distinctive for each strains are depicted as symbols, with filled symbols indicating presence and empty symbols indicating absence of a given gene (as indicated in the legend). The entire data sets on total, protein and PIA biofilm formation can be found in **Supplementary Figure S1**. The means were calculated from three biological replicates run in duplicates. Statistical analysis was performed using one-way ANOVA by employing the GraphPad Prism software package. ns: *P* = 0.1234; *P = 0.0332; ****P < 0.0001.

As mentioned above, we previously reported that *S. aureus* Rd11 carrying pAFS11 does not produce biofilm, nor does *S. aureus* RN4220 into which the plasmid was transformed, suggesting that *ica*_pAF_ on the plasmid might be inactive (Feßler et al., 2017). To challenge this hypothesis, we cloned the entire *ica*_pAF_ from pAFS11 onto the shuttle vector pRB473 (resulting in plasmid p*ica*_pAF_) to enable ready genetic manipulation of the locus. As a first step, we deleted the *icaR*_pAF_ coding region from the vector, yielding plasmid p*ica*_pAF__Δ*icaR*. Both plasmids (with or without *icaR*_pAF_) were transformed into *S. aureus* RN4220 as recipient strain and biofilm assays were performed with the constructs and corresponding wild types (**Figure 2**). The biofilm assays allow to detect total biofilm formation as well as to differentiate between PIA and protein matrix-mediated biofilm production (see material and methods for details). We display here (and in the following figures) solely the data for PIA-mediated biofilm formation (**Figures 2**–**5**). The entire data sets on total, protein and PIA biofilm formation can be found in **Supplementary Figure S1**. The assays confirmed the PIA biofilm-negative phenotype of *S. aureus* Rd11 and revealed that the *S. aureus* RN4220 wild type is a weak, but detectable PIA biofilm producer. Upon acquisition of pAFS11, PIA biofilm formation of RN4220 did not increase, but on the contrary was even slightly reduced, although this reduction was statistically not significant (+pAFS11, **Figure 2**). The same phenomenon occurred when *S. aureus* RN4220 was transformed with plasmid p*ica*_pAF_, carrying the entire *ica*_pAF_ locus from pAFS11 (+p*ica*_pAF_, **Figure 2**). In contrast, however, PIA biofilm formation of RN4220 massively increased when the *icaR*_pAF_ gene was deleted from the *ica*_pAF_ copy on the vector (*+*p*ica*_pAF__Δ*icaR*, **Figure 2**). In the *S. aureus* RN4220 +p*ica*_pAF__Δ*icaR* strain, PIA biofilm levels even exceeded that of the *S. epidermidis* RP62A positive control and were much higher than in the RN4220 wild type, suggesting that the *ica*_pAF_ copy on the vector contributes to PIA production, but only when the IcaR_pAF_ repressor is absent. We conclude from this that pAFS11 may exert its negative effect on *S. aureus* PIA biofilm formation most likely *via* IcaR_pAF_ which also seems to negatively influence the *ica* locus on the RN4220 chromosome (hereinafter referred to as *ica*_RN_).

### The *ica* Locus on pAFS11 Is Inactive Due to Efficient IcaR Repression

The data obtained so far strongly suggest that the *ica*_pAF_ locus is functional and capable to enable PIA synthesis. However, by our initial experimental set-up (*i.e.* by employing the *S. aureus* RN4220 wild type with an intact chromosomal *ica*_RN_ locus) it was difficult to distinguish between *ica*_pAF_- and *ica*_RN_-derived PIA production. Therefore, we constructed a markerless *ica*_RN_ deletion mutant in RN4220 *via* allelic replacement, and transformed the resulting RN4220 Δ*ica* strain with plasmids pAFS11, p*ica*_pAF_ and p*ica*_pAF__Δ*icaR.* Biofilm assays with the constructs confirmed loss of PIA production in the RN4220 Δ*ica* deletion mutant (**Figure 3A**). Providing the mutant with an entire *ica*_pAF_ locus either on plasmid pAFS11 or p*ica*_pAF_ did not result in biofilm formation (**Figure 3A**). However, biofilm formation was triggered and highly significantly increased, when the *icaR*_pAF_ repressor-encoding gene was deleted from the *ica*_pAF_ locus (+p*ica*_pAF__Δ*icaR*, **Figure 3A**), indicating that the *icaADBC*_pAF_ genes of *ica*_pAF_ are indeed able to mediate PIA biofilm formation, once IcaR_pAF_-dependent repression is alleviated. To further corroborate this assumption, we monitored transcription of *icaA*_pAF_ and *icaR*_pAF_ by qRT-PCR in the various constructs. In strain RN4220 Δ*ica*, transformed with either pAFS11 or p*ica*_pAF_, weak *icaA*_pAF_ transcription was detectable (**Figure 3B**). Upon deletion of *icaR*_pAF_ from the plasmid (+p*ica*_pAF__Δ*icaR*, **Figure 3B**), *icaR*_pAF_ transcription was no longer detectable (as expected) and *icaA*_pAF_ transcription levels massively increased (*i.e*. 200-fold) compared to the intact *ica*_pAF_ copy (+p*ica*_pAF,_ **Figure 3B**). These findings are in agreement with the biofilm test results. From the combined data we conclude that (i) the *ica*_pAF_ locus on pAFS11 is functionally fully intact and (ii) *icaADBC*_pAF_ operon transcription is efficiently repressed by its cognate IcaR_pAF_ repressor.

### IcaR From pAFS11 Represses the *ica* Locus in the *S. aureus* Chromosomal DNA and *vice versa*

Our initial experiments with wild type *S. aureus* RN4220 indicated that IcaR_pAF_ may also inhibit *icaADBC*_RN_ expression on the RN4220 chromosome (**Figure 2**). To substantiate this hypothesis, we constructed another set of plasmids carrying (i) the *ica* locus from RN4220 (p*ica*_RN_), (ii) the *ica* locus from RN4220 lacking *icaR*_RN_ (p*ica*_RN__Δ*icaR*_RN_) and (iii) the *ica* locus from RN4220 where we exchanged *icaR*_RN_ from RN4220 with *icaR*_pAF_ from pAFS11 (p*ica*_RN__Δ*icaR*_RN__*icaR*_pAF_). All plasmids were transformed into the RN4220 Δ*ica* mutant background and the resulting strains were analyzed for their ability to form PIA biofilm (**Figure 4A**). Complementation of RN4220 Δ*ica* with its own *ica*_RN_ locus restored PIA-mediated biofilm formation, and upon *icaR*_RN_ repressor gene deletion, PIA biofilm production significantly increased, demonstrating functionality of the vector-borne *ica*_RN_ locus, including *icaR*_RN_-mediated regulation (**Figure 4A**). Accordingly, qRT-PCR analysis confirmed that IcaR_RN_ efficiently represses transcription of its cognate *icaADBC*_RN_ operon (**Figure 4B**). We then asked the question whether or not expression of the chromosomal *icaADBC*_RN_ operon can undergo control by the foreign IcaR_pAF_ repressor from pAFS11. Thus, we performed biofilm tests and quantitative transcription analyses with vector p*ica*_RN__Δ*icaR*_*icaR*_pAF_, in which *icaADBC*_RN_ was combined with the *icaR*_pAF_ gene from pAFS11. As shown in **Figure 4**, presence of *icaR*_pAF_ significantly diminished PIA production and transcription of the *icaADBC*_RN_ operon, suggesting the capability of IcaR_pAF_ to control the *icaADBC*_RN_ copy from RN4220 (**Figure 4**). *Vice versa*, we next investigated, if IcaR_RN_ from RN4220 can influence *icaADBC*_pAF_ from pAFS11. For this purpose, we additionally constructed vector p*ica*_pAF__Δ*icaR*_pAF__*icaR*_RN_ which was transformed into the RN4220 Δ*ica* mutant background. Biofilm testing revealed a highly significant reduction of PIA production when *icaADBC*_pAF_ was combined with *icaR*_RN_, suggesting that IcaR from *S. aureus* RN4220 is indeed able to repress the *icaADBC*_pAF_ genes from pAFS11 (**Figure 4C**).

### A Palindrome Sequence in the *icaA*_pAF_ Upstream Region Is Required for IcaR-Mediated Biofilm Repression

The data obtained so far demonstrate that the pAFS11- and RN4220-derived IcaR repressors, which differ on amino acid level (**Figure 1D**), are able to control the *icaADBC* operon of the respective other *ica* locus copy. To understand the molecular prerequisites for the IcaR interactions with their DNA targets, we focused on the nucleotide sequence constraints known to be involved in IcaR binding. IcaR was previously shown to bind as a dimer to a specific palindrome sequence (ACCTANCTNNC/GNNAGNTAGGT) present in the *icaA* operator of *S. epidermidis* (Jeng et al., 2008). This palindrome is 22 nucleotides long and contains six mismatches (22,6) (**Figure 5A**, top). Of note, the sequence is highly conserved and is also present in the *S. aureus icaA* promoter region (**Figure 5A**, top). Surprisingly, although IcaR from *S. aureus* is clearly able to control PIA production from pAFS11, the *S. aureus*-like palindrome sequence stretch lacks in *ica*_pAF_ (**Figure 5A**, bottom). Instead, the *icaA*_pAF_ upstream region displays two other palindromes which differ at the nucleotide level from that of the known *S. aureus/S. epidermidis* recognition site. Thus, palindrome A (TNAAAATNNTA/TANNATTTTNA) is 22 nucleotides long and harbors six mismatches (22,6), while palindrome B (CNAACNANC/GNTNGTTNG) consists of 18 nucleotides with six mismatches (18,6) (**Figure 5A**, bottom). To investigate the putative involvement of these palindromes in IcaR function, we mutated either palindrome A or B in (i) a plasmid carrying the whole *ica*_pAF_, (ii) in an *ica*_pAF_ plasmid with an *icaR*_pAF_ deletion as well as (iii) in an *icaR*_pAF_ deletion vector complemented with *icaR*_RN_. The plasmids were again transformed into the RN4220 Δ*ica* mutant background and analyzed for PIA-mediated biofilm formation. **Figure 5B** demonstrates that an altered palindrome A sequence did not influence *icaR*-mediated biofilm control. Thus, upon palindrome A mutation, PIA-mediated biofilm production remained repressed in p*ica*_pAF(A_***_)_, indicating that IcaR_pAF_ does not require this nucleotide sequence stretch for action. As expected, PIA production was derepressed when *icaR*_pAF_ was lacking in the palindrome A mutant (+p*ica*_pAF(A_***_)__Δ*icaR*, **Figure 5B**). Most importantly, however, *icaR*_RN_ was still able to completely downregulate PIA-mediated biofilm production in this construct, confirming that palindrome A is not an interaction site for IcaR, neither for IcaR proteins derived from pAFS11 nor from RN4220 (**Figure 5B**). In contrast, mutation of palindrome B had a profound impact on biofilm regulation by IcaR. Firstly, PIA production was found to be deregulated and increased in an *ica_pAF_* construct carrying an altered palindrome B nucleotide sequence (+p*ica*_pAF(B_***_)_), suggesting that control by the cognate IcaR_pAF_ is significantly impaired when integrity of this sequence stretch is disturbed (**Figure 5B**). Moreover, PIA-mediated biofilm production further increased in a palindrome B mutant in which *icaR*_pAF_ was deleted (+p*ica*_pAF(B_***_)__Δ*icaR*, **Figure 5B**), which speaks in favor of residual IcaR_pAF_ repressor activity in the palindrome B mutant. Finally, when providing the mutant with IcaR_RN_ from RN4220, biofilm repression was partially, but not fully restored which (again) is in good agreement with residual IcaR repression in the palindrome B mutant. Together, the combined data strongly suggest that IcaR from both pAFS11 and RN4220 require an intact palindrome B (but not palindrome A) for unfolding their repressor activity on the *ica*_pAF_ locus.

## Discussion

Acquisition of mobile genetic elements (MGEs) is often beneficial for bacteria by providing novel metabolic and resistance traits. However, MGE carriage may also come at considerable cost for the recipient bacterial cell (Slater et al., 2008; San Millan and MacLean, 2017). Thus, resources will be required to replicate and maintain MGEs (*e*.*g.* plasmids) on which beneficial genes are located and their (inappropriate) expression may impose a metabolic burden, resulting in reduced fitness and competitiveness of MGE-bearing bacterial cells (Baltrus, 2013). Accordingly, bacteria have evolved sophisticated mechanisms to control both MGE uptake and maintenance as well as expression of horizontally acquired genes (Brantl, 2014; Kwong et al., 2017; Firth et al., 2018). In case of plasmid-mediated HGT, this often involves a regulatory crosstalk between chromosomal factors and the newly acquired plasmid (Huang et al., 1990; Charles and Nester, 1993; Baños et al., 2009). Interestingly, these control networks are not unidirectional and there is growing evidence to suggest that plasmids are able to influence chromosomal gene expression as well in a wide range of bacterial species [recently reviewed in (Vial and Hommais, 2020)]. In the study presented here, we extend these examples by the Gram-positive pathogen *S. aureus* and reveal that horizontally acquired and core genome genes have the capacity to mutually influence each other in this organism. Thus, we demonstrate that a transcription factor (*i.e.* IcaR), located on a large multi-resistance plasmid, is able to target a pathogenicity factor (*i.e*. *icaADBC*-mediated PIA biofilm formation) from the *S. aureus* core genome. *Vice versa*, the IcaR homolog from the *S. aureus* core genome was found to be able to silence transcription of plasmid-borne *icaADBC* genes, creating a bi-directional regulatory crosstalk between plasmid- and chromosomally encoded factors that eventually hindered metabolically costly PIA-mediated biofilm formation.

PIA consists of N-acetyl-glucosamines (GlcNac) molecules, and ample sugar and energy supplies are fuelled into GlcNac synthesis to provide the building blocks of the exopolysaccharide. Accordingly, *ica* gene expression is intimately linked to central carbon flux control and energy balance (Vuong et al., 2005; Seidl et al., 2008; Zhu et al., 2009; Sadykov et al., 2011; Lindgren et al., 2014) which also involves the action of non-coding RNAs to appropriately adjust metabolic patterns (Rochat et al., 2018; Bronesky et al., 2019; Marincola et al., 2019; Schoenfelder et al., 2019). Presence of two fully functional *ica* gene clusters in strain *S. aureus* Rd11 is likely to represent a major metabolic challenge and the observed downregulation of PIA production in this strain makes sense to prevent metabolic overload. Paradoxically, it is just the additionally acquired *ica*_pAF_ locus copy on plasmid pAFS11 that mediates a biofilm-negative phenotype. Indeed, acquisition of pAFS11 or the *ica*_pAF_ locus alone abolished PIA biofilm formation in *S. aureus* (**Figure 2**). This effect is accomplished through the IcaR_pAF_ repressor which can also target the chromosomal *ica* locus copy (**Figure 4**). Moreover, the tight self-control of the *ica*_pAF_ copy on pAFS11 by its cognate IcaR_pAF_ repressor further contributes to a biofilm-negative phenotype (**Figures 2**, **3**). Remarkably, *icaADBC*_pAF_ expression can be additionally repressed by Ica_RN_ from the core genome (**Figure 4C**). Thus, although being different from the canonical IcaR recognition site known from *S. epidermidis* and *S. aureus* (Jeng et al., 2008), Ica_RN_ interacts with a palindrome sequence present in the upstream region of *icaADBC*_pAF_ (palindrome B in **Figure 5A**), revealing a certain flexibility of IcaR-like proteins in DNA target selection (**Figure 5**).

Phylogenetic analyses revealed that the two *ica* loci in Rd11 differ both on nucleotide and protein sequence levels and are only distantly related to each other (**Figure 1**). Thus, the horizontally acquired *ica*_pAF_ copy on pAFS11 is likely to originate from an unknown bacterium for which the soil and animal dwelling species *S.* *sciuri* [recently re-classified as *M. sciuri* (Madhaiyan et al., 2020)] might represent a putative candidate (Feßler et al., 2017). But clearly more detailed investigations will be required to substantiate this hypothesis. Interestingly, inhibition of core genome-encoded exopolysaccharide production by plasmids seems to be a common theme in the bacterial world. Thus, in nitrogen-fixing *Rhizobium tropici*, exopolysaccharide production was found to be downregulated by the NrcR transcription factor, encoded on an acquired plasmid (Del Cerro et al., 2016), and in the nosocomial pathogen *Acinetobacter baumanii*, PNAG (PIA) production was described to be diminished upon acquisition of a multi-resistance plasmid (Venanzio et al., 2019). In the latter case, this large conjugative multiresistance plasmid facilitates its own transmission by downregulating chromosomally encoded type-6-secretion systems (T6SS) that usually hamper HGT (Venanzio et al., 2019). Together with the effect on PNAG production, the *A. baumanii* plasmids represent interesting examples for a plasmid-chromosome regulatory crosstalk that influences simultaneously both virulence and resistance traits.

LA-MRSA lineages of the clonal complex CC398 (to which strain Rd11 belongs to) can thrive in very different habitats (*e.g.* animals, humans, environment etc.) where they are exposed to numerous stress conditions. It is tempting to speculate that PIA biofilm formation, which is an important factor in *S. aureus* pathogenesis and survival (Fluckiger et al., 2005; Kropec et al., 2005), might become a selection advantage at some stage. In this respect, it is an interesting question by which mechanism(s) biofilm formation could be restored in the Rd11 isolate. Plasmid pAFS11 displays a striking mosaic structure and has most likely arisen by recombination of genes from various origins. Assuming that *ica*_pAF_ locus integration into pAFS11 and acquisition of the plasmid by *S. aureus* is an evolutionarily recent event, it is conceivable that control of pAFS11-encoded gene expression is not fully integrated into the regulatory network of the *S. aureus* Rd11 recipient (yet). One obvious possibility to (re)gain a biofilm positive phenotype would be (spontaneous) mutation and inactivation of *icaR*_pAF_ on pAFS11. At first sight, this idea seems to be contradictory to our experimental findings showing that IcaR_RN_ has the potential to take over and replace the lacking IcaR_pAF_ activity. This mutual IcaR replacement seems to work particularly well, when both factors (*icaR* and *icaADBC*) are on the same replicon and are probably in an appropriate stoichiometric repressor/DNA equilibrium (**Figure 4C**). In agreement with this assumption, the IcaR_RN_ repressor effect was found to be less efficient when a single *icaR*_RN_ copy resides on the chromosome and *icaADBC*_pAF_ is located on a (multi-copy) plasmid (**Figure 2**). Here, *icaR*_pAF_ deletion on the vector enabled PIA production, and it is reasonable to suggest that the insufficient repressor activity of Ica_RN_ in this experimental set-up might be associated with the copy number of the *ica*_pAF_-bearing vector which increased the number of DNA targets for the IcaR_RN_ repressor. At the present stage of experimental work this is mere speculation. However, recent research demonstrates that available IcaR protein amounts are critical for the appropriate control of *icaADBC* transcription, and multiple *cis*- and *trans*-acting factors have been identified that target *icaR* mRNA molecules to fine-tune their translation (Ruiz de los Mozos et al., 2013; Rochat et al., 2018; Bronesky et al., 2019; Lerch et al., 2019; Schoenfelder et al., 2019). Plasmid copy number effects may have the potential to interfere with this delicate repressor/DNA target balance. In this respect, the data obtained with *ica*_pAF_-bearing plasmids in the *ica*_RN_ locus proficient *S. aureus* RN4220 background may reflect very well the natural situation in pAFS11 carrying isolates (**Figure 2**). Thus, from an evolutionary point of view, mutational inactivation of *icaR*_pAF_ on the pAFS11 plasmid would make sense to readily enable PIA production. The same effect could be achieved by acquiring mutations in the *ica*_pAF_ palindrome B sequence, which represents the target site of both the IcaR_pAF_ and IcaR_RN_ proteins. Indeed, at least for *S. aureus* core genome *ica* loci, mutations in *icaA* upstream regions were previously described. The mutations had direct consequences for PIA production and occurred both *in vitro* and *in vivo*, indicating that the *icaA* promoter region undergoes selection and is a suitable target to modulate PIA production (Jefferson et al., 2003; Schwartbeck et al., 2016). It will be interesting to explore if the *ica*_pAF_ locus on pAFS11 might become subject to mutational variation. Long-term *in vitro* passage experiments together with surveillance of the evolution of pAFS11-like plasmids in field studies will be suitable approaches to give answers to how control of plasmid-borne *ica* locus expression will be integrated into the regulatory network of *S. aureus*.

## Data Availability Statement

The original contributions presented in the study are included in the article/**Supplementary Material**. Further inquiries can be directed to the corresponding authors.

## Author Contributions

GM, AF, SS, and WZ: conceived and designed the experiments. GM, GJ and A-KK performed the experiments. GM, GJ, A-KK, FW and WZ analyzed the data. GM and WZ wrote the manuscript. All authors contributed to the article and approved the submitted version.

## Funding

The study was supported by the German Research Council (DFG) through grant ZI665/3-1 as well as by the German Federal Ministry of Education and Research (BMBF), grant numbers 01KI1727E, 01KI1727D, 01KI2009D as part of the Research Network Zoonotic Infectious Diseases as well as grant number 16GW0297 within the program ‘Target validation for pharmaceutical drug development’. This publication was supported by the Open Access Publication Fund of the University of Wuerzburg.

## Conflict of Interest

The authors declare that the research was conducted in the absence of any commercial or financial relationships that could be construed as a potential conflict of interest.
