# Supplementary material for: Plasmid-Chromosome Crosstalk in Staphylococcus aureus: A Horizontally Acquired Transcription Regulator Controls Polysaccharide Intercellular Adhesin-Mediated Biofilm Formation
Source: Front Cell Infect Microbiol. 2021 Mar 22;11:660702. doi: 10.3389/fcimb.2021.660702 (PMC8019970; doi:10.3389/fcimb.2021.660702)
Supplement: Supplementary file 1 [file Image_1.pdf]

Supplemental Figure S1

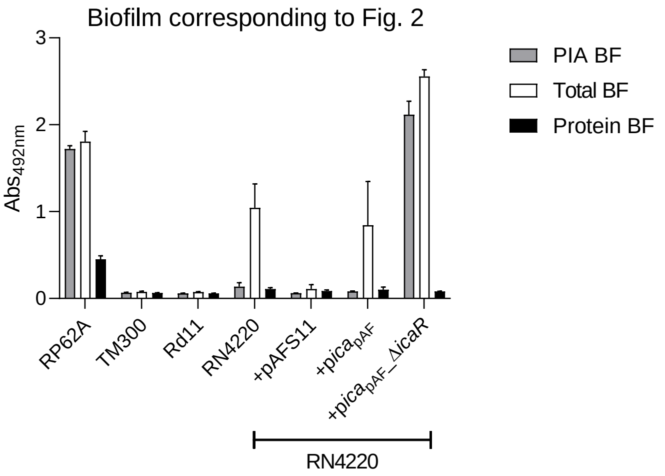

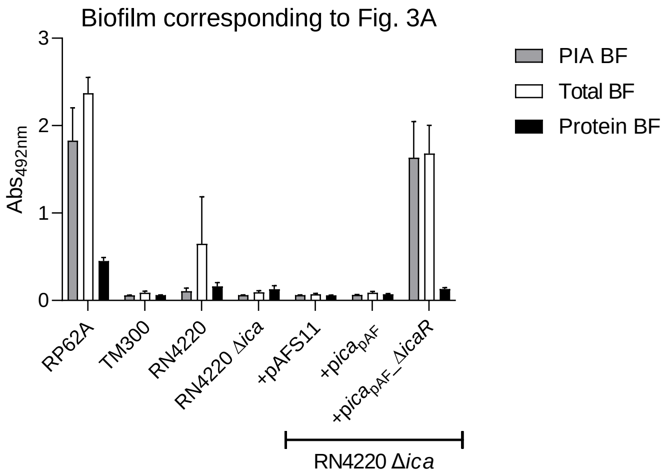

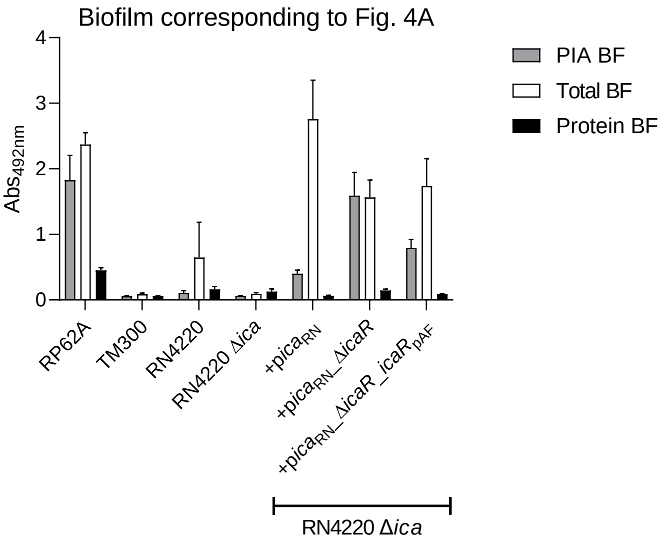

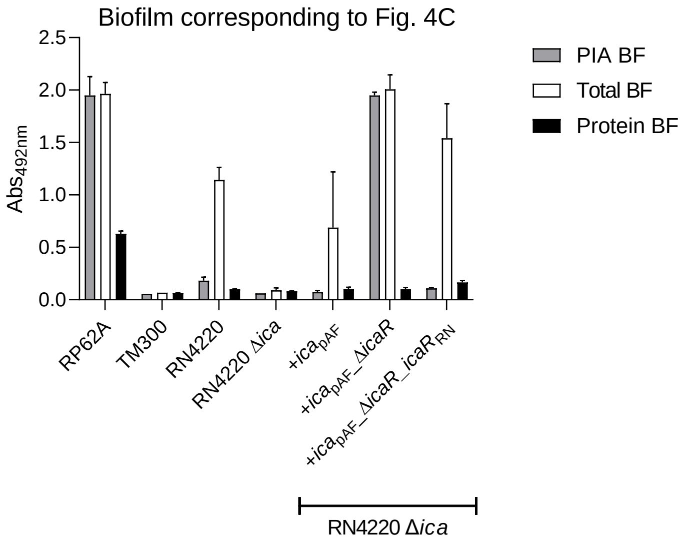

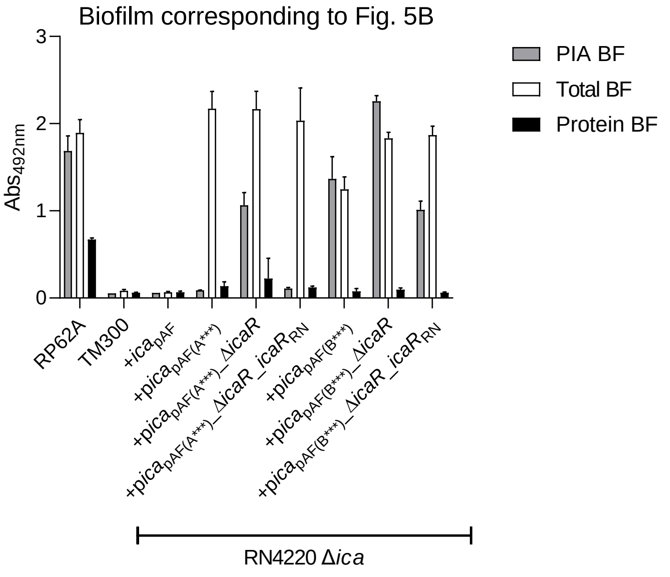

Supplemental Figure S1. Entire data sets on total, protein and PIA biofilm formation corresponding to Figures 2, 3, 4 and 5. Biofilm formation was assessed using crystal violet staining in 96-well polystyrene tissue culture plates upon static growth at 30 °C in TSB supplemented with 4 % NaCl. RP62A and TM300 served as positive and negative controls respectively. In addition to total biofilm mass, protein- and PIA-mediated biofilm proportions were determined by sodium-periodate and proteinase K treatments, respectively, as described in Material and Methods. The means were calculated from three biological replicates run in duplicates.
